# Identification of the Neutralizing Epitopes of Merkel Cell Polyomavirus Major Capsid Protein within the BC and EF Surface Loops

**DOI:** 10.1371/journal.pone.0121751

**Published:** 2015-03-26

**Authors:** Maxime J. J. Fleury, Jérôme T. J. Nicol, Mahtab Samimi, Françoise Arnold, Raphael Cazal, Raphaelle Ballaire, Olivier Mercey, Hélène Gonneville, Nicolas Combelas, Jean-Francois Vautherot, Thierry Moreau, Gérard Lorette, Pierre Coursaget, Antoine Touzé

**Affiliations:** 1 L’UNAM Université, Groupe d’Etude des Interactions Hôte-Pathogène, UPRES EA 3142, Université d'Angers, Angers, France; 2 UMR INRA 1282, Virologie et Immunologie Moléculaire, Faculté des Sciences Pharmaceutiques, Université François Rabelais, PRES Centre-Val de Loire Université, Tours, France; 3 CHRU de Tours—Hôpital Trousseau, Service de Dermatologie, Tours, France; 4 UMR INRA 1282, Biologie des virus aviaires, INRA Nouzilly, France; 5 UMR INSERM 1100, Mécanismes Protéolytiques dans l'Inflammation, Faculté de Médecine, Université François Rabelais, PRES Centre-Val de Loire Université, Tours, France; 6 Faculté des Sciences Pharmaceutiques, Université François Rabelais, PRES Centre-Val de Loire Université, Tours, France; New York State Dept. Health, UNITED STATES

## Abstract

Merkel cell polyomavirus (MCPyV) is the first polyomavirus clearly associated with a human cancer, i.e. the Merkel cell carcinoma (MCC). Polyomaviruses are small naked DNA viruses that induce a robust polyclonal antibody response against the major capsid protein (VP1). However, the polyomavirus VP1 capsid protein epitopes have not been identified to date. The aim of this study was to identify the neutralizing epitopes of the MCPyV capsid. For this goal, four VP1 mutants were generated by insertional mutagenesis in the BC, DE, EF and HI loops between amino acids 88-89, 150-151, 189-190, and 296-297, respectively. The reactivity of these mutants and wild-type VLPs was then investigated with anti-VP1 monoclonal antibodies and anti-MCPyV positive human sera. The findings together suggest that immunodominant conformational neutralizing epitopes are present at the surface of the MCPyV VLPs and are clustered within BC and EF loops.

## Introduction

Polyomaviruses are known to infect mammals and birds. Thirteen human polyomavirus have been identified to date including BKPyV [1], JCPyV [2], KIPyV [3], WUPyV [4], Merkel cell polyomavirus (MCPyV) [5], HPyV6 and HPyV7 [6], TSPyV [7], HPyV9 [8], MWPyV [9–11], STLPyV [12], HPyV12 [13] and NJPyV [14]. MCPyV has been associated with Merkel cell carcinoma (MCC) and is now recognized as a 2A carcinogen by IARC [15].

MCC is a relatively rare but aggressive skin cancer with a mortality rate higher than melanoma. MCC is rarely observed in people younger than 50 years of age, and the risk of developing this cancer increases with age, immunodeficiency and sun exposure [16–18]. MCPyV was identified in 2008 as the causative agent of the majority of MCC [5,19]. Viral particles are not produced in MCC tumor cells [20,21] and the specific cells in which MCPyV infectious viral particles are produced have not yet been identified.

Polyomaviruses are small naked DNA viruses and their icosahedral capsid of about 45 nm in diameter is constituted of VP1, VP2 and VP3 proteins, and encapsidate a double-stranded circular DNA of about 5 kbp. The minor capsid proteins VP2 and VP3 are sequestered within the shell of the capsid formed by the VP1 protein [22]. As for SV40 polyomavirus, each VP1 monomer is composed of two antiparallel b-sheets, which together form a b-sandwich with jelly-roll topology [23]. The two sheets consist of strands that support extensive loops, named BC, DE, EF and HI, exposed at the surface and sides of the pentamer. These loops are the most variable parts of VP1 sequences.

Serological studies have shown that, as observed for other human polyomaviruses, most adults have had prior exposure to MCPyV [20,24–29]. The nature of the epitopes that elicit antibodies against the viral capsid is unknown. Immunization of mice with MCPyV VP1 virus-like particles (VLPs) induces high titers of antibodies [20,25] which have been shown to be neutralizing [20] as anti-MCPyV VP1 monoclonal antibodies [30].

The antibody response against small naked DNA virus is typically generated against epitopes exposed at the surface of the VLPs [31–34]. However, the nature of the epitopes that elicit antibodies to polyomavirus capsid proteins is largely unknown except for SV40, the epitopes of which have been mapped using monoclonal antibodies and replicative mutants in the BC and EF loops [35].

In order to identify the major MCPyV VP1 conformational epitopes, we investigated the reactivity of wild-type and four VP1 protein insertional mutants against a panel of anti-MCPyV VP1 monoclonal antibodies (mAbs) and anti-MCPyV positive human sera.

## Materials and Methods

### Generation and characterization of MCPyV VP1 mutants

In addition to MCPyV [21,25], SV40 [36], BKPyV [37], LPyV [25], HPyV6, HPyV7, HPyV9 and TSPyV [38] VP1 VLPs, four MCPyV VP1 insertional mutants were also produced in insect cells using recombinant baculoviruses. For this purpose, MCPyV VP1 gene mutants were generated by SOE-PCR using the MKT21 sequence as template (FM864207.1). The StreptagII motif (WSHPQFEK) coding sequence was inserted in the predicted surface exposed loops (after S88 of BC, after H150 of DE, after T189 of EF, after T296 of HI) using the MKT21 sequence model generated by Swiss-Model (http://swissmodel.expasy.org/) and the 1SVA pdb file as template. PCR fragments representing the 5’ and 3’ parts of the VP1 gene were obtained in an initial step of 6 cycles (94°C 30s, 50°C 30s, 72°C 2 min) using 5’fullVP1 and 3’ loop primer and 5’ loop primer and 3’ full VP1, respectively (Table 1). Then 5’ and 3’ fragments were assembled in a 6 cycle second PCR step (94°C 30 s, 50°C 30 s, 72°C 2 min 30 s). PCR products were cloned by TA cloning into the pCR2.1TOPO plasmid (Invitrogen, Fischer, Illkirch, France). The presence of the StreptagII motif sequence and absence of unwanted mutagenesis was verified by sequencing (MWG Biotech, Ebersberg, Germany). Modified VP1 genes were then cloned between the *BamHI* and *HindIII* restriction sites of the pFastBac1 plasmid (Invitrogen) to generate the different recombinant baculoviruses. Recombinant baculoviruses were generated using the Bac to Bac technology (Invitrogen).

**Table 1:** Sequence of primers used for generation of MCPyV VP1 mutants^a^

Name	5’→3’	Restriction site
5’ full VP1	*GGATCC*CCTGAATTACAAGTAATTGAAGATGGCACC	*Bam*HI
3’ full VP1	*AAGCTT*CTGAATAGGAATGCATGAAATAATTCTCAT	*Hind*III
5’ BC loop	TGGAGCCATCCGCAATTTGAAAAGTCTCCAGATCAGCCCATCAAGG	
3’ BC loop	CTTTTCAAATTGCGGATGGCTCCATGATCCCTTTGGCTGCAGGTCATAAG	
5’ DE loop	TGGAGCCATCCGCAATTTGAAAAGGATTACGGTGCTGGTATTCC	
3’ DE loop	CTTTTCAAATTGCGGATGGCTCCAATGAACTCTTTTCATGTCCC	
5’ EF loop	TGGAGCCATCCGCAATTTGAAAAGACAAATGGTGGGCCTATTACAATTG	
3’ EF loop	CTTTTCAAATTGCGGATGGCTCCAAGTTTTTGGATACTCAGTCTGGTAATC	
5’ HI loop	TGGAGCCATCCGCAATTTGAAAAGAGTGGAAAAATGGCTCTTCATGGG	
3’ HI loop	CTTTTCAAATTGCGGATGGCTCCAGGTTTTAAACAGAAACCCCACTATG	

^a^ Restriction site sequences are italicized

For the production of VLPs, Sf21 insect cells maintained in SF900II serum-free medium (Invitrogen) at 27°C were infected with the different recombinant baculoviruses. VLPs were purified as described previously and the presence of VLPs was analyzed by transmission electron microscopy [25]. Exposure of the StreptagII motif was searched by ELISA using a mouse anti-StreptagII monoclonal antibody conjugated to HRP (Novagen, VWR, Strasbourg, France). Microplates (Maxisorp, Nunc) were coated with 200 ng per well of VLPs or PBS and incubated at 4°C overnight. Wells were then blocked with PBS supplemented with 1% FCS for 1 h at 37°C. Duplicate wells (two tests and one control) were incubated with anti-StreptagII-HRP monoclonal antibody diluted 1:1000 in dilution buffer (PBS 5X, 1% Tween, 10% FCS) for 1 h at 37°C. After four washes, 100 μl of a solution containing 0.4 mg/ml o-phenylene-diamine and 0.03% hydrogen peroxide in 25 mM sodium citrate and 50 mM Na2HPO4 was added. The reaction was stopped after 30 min with 100 μl 4 N H2SO4 and absorbance was read at 490 nm. A positive result was recorded when the difference in OD between test and control wells was greater than 0.20. The results are the means of three determinations.

### Production of monoclonal antibodies

Six week-old female Balb/C mice (IFFACredo, St. Germain l’Arbresle, France) were immunized by intrapodal injection with purified MCC350 aggregates or MKT21 VLPs (50 μg) emulsified in Quil A (50 μg), conducted as previously described with minor modifications [39]. After 12 days, each mouse was boosted with the same preparation. Three days later, mice were sacrificed and popliteal lymph nodes were collected. Lymphocytes were collected by perfusing the lymph node with RPMI 1640-Glutamax medium (Invitrogen). Cells were washed once in RPMI and centrifuged at 300 g for 5 min and mixed with Sp2/O myeloma cells at a ratio of 1:5. Cells were then collected by centrifugation at 300 g for 5 min and fusions were performed as previously described [40]. Hybridoma culture supernatants were screened by ELISA for antibody reactivity to intact MCPyV VP1 VLPs and to dissociated VLPs obtained by treatment with 0.1 M carbonate buffer (pH 10.6) and 0.01 M dithiothreitol (DTT) in PBS for 30 min at 37°C. Subcloning was performed by end-point dilutions in 96-well plates and reactive subclones were isotyped using the mouse mAb isotyping kit according to the recommended procedure (Sigma-Aldrich). To characterize the type specificity of these mAbs, ELISAs were performed with MCPyV, SV40, LPyV, HPyV6, HPyV7, HPyV9, TSPyV and BKPyV VP1 VLPs. For ELISA, microplates (Maxisorp, Nunc) were coated with 200 ng of VLPs per well of and incubated at 4°C overnight and then blocked with PBS supplemented with 1% FCS for 1 h at 37°C. Duplicate wells (two tests and one control) were incubated with hybridoma culture supernatant diluted 1:3 in dilution buffer (PBS 5X, 1% Tween, 10% FCS) for 1 h at 37°C. The plates were washed four times and probed with peroxidase-conjugated goat anti-mouse Ig Fc (Sigma-Aldrich; 1:5,000 dilution) for 1 h at 37°C. After four washes, colorimetric revelation was performed as above. A positive result was scored when the difference in OD between test and control wells was greater than 0.20. The data presented are the means of three determinations.

All animal procedures were performed according to approved protocols and in accordance with the recommendations for the proper use and care of laboratory animals, and experiments were approved by the Regional Animal Ethics Committee (Approval ID CL2006-048, Comité Régional d'Ethique en Matière d'Expérimentaion Animale Centre-Limousin).

### Generation of MCPyV pseudovirions and detection of anti-MCPyV neutralizing antibodies

MCPyV pseudovirions were produced in human embryonic kidney-derived 293FT cells. The 293FT cell line (Invitrogen) is a fast growing variant of the 293 cell line that stably expresses SV40 TAg and the neomycin resistance gene from pCMVPORT6AT.neo plasmid. 293FT cells were grown in Dulbecco’s modified Eagle’s Medium, supplemented as above, plus 1% non-essential amino acids and 500 mg/ml G418 (Invitrogen). Cell lines were grown at 37°C in a humidified atmosphere with 5% CO2. These cells were co-tranfected with expression plasmids carrying codon-modified versions of MCPyV VP1 (pwM) and VP2 genes (ph2m) of MCPyV isolate 339 [41] (generous gift of Chris Buck, NCI) and the pGL4 plasmid as luciferase expression reporter (Promega). Transfected cells were then cultured for 3 days in complete Dulbecco’s modified Eagle’s medium (Invitrogen, DMEM supplemented with 10% FCS, 100 IU/ml penicillin, 100 μg/ml streptomycin and 250 μg/ml hygromycin B). Pseudovirions present in the nuclear fraction of 293FT (MCPyV PsV stock) were titered by measuring their end-point luciferase gene transduction capacities on COS-7 cells (African green monkey kidney cells, ATCC CRL-1651). For the detection of neutralizing antibodies, COS-7 cells (10^4^/well) were seeded in 96-well plates (TPP, Dutscher, Brumath, France). COS-7 cells were grown in Dulbecco’s modified Eagle’s Medium (Invitrogen) supplemented with 10% heat-inactivated fetal calf serum (FCS), 100 IU/ml penicillin, and 100 μg/ml streptomycin and 1 mM sodium pyruvate. After 24h incubation at 37°C, cells were washed twice before addition of pseudovirion/antibody mixture. The amount of pseudovirions was adjusted to obtain a relative luciferase activity of 0.2 RLU (Luminoskan Ascent, Thermo Scientific, Courtaboeuf, France). Twenty five microliters of diluted pseudovirions were mixed with 25 μl of hybridoma supernatants at a final dilution of 1:3 in incomplete DMEM medium or human sera at a final dilution of 1:1000. After 1h incubation at 37°C, the mixture was added to the wells and plates were incubated for 3 h at 37°C. Then, the mixture was removed, 100 μl of DMEM-FCS were added and, after incubation at 37°C for 48 h, the luciferase gene expression was measured (Luciferase reporter gene assay with constant light signal, Roche Molecular Biochemical, Meylan, France). The results were expressed as the percentage of inhibition of luciferase activity [40,42]. The data presented are the means of 3 determinations performed in duplicate. Antibodies were considered to be neutralizing for inhibition greater than 80%.

For investigation of post-attachment neutralization, pseudovirions were bound to cells for 1 h at 4°C. Unbound virions were removed by washing cells with serum-free DMEM and then antibodies diluted 1:3 in DMEM-FCS were added to a total volume of 50 μl. After 1h at 37°C, the antibodies were removed, then 100 μl of DMEM-FCS were added and after incubation for 48 h the luciferase activity was measure as above.

### Reactivity of MCPyV mAbs and human antibodies against MCPyV VLP mutants

In order to locate the epitopes of the MCPyV VP1, the reactivity of mAbs and human antibodies was analyzed using the MCPyV wt VP1 VLPs and the four mutants with insertion within the BC, DE, EF and HI VP1 loops. ELISAs were performed as described above for this purpose. Hybridoma culture supernatants were diluted 1:3 and human anti-MCPyV positive sera were diluted 1:1000 in dilution buffer (PBS 5X, 1% Tween, 10% FCS) for 1 h at 37°C. Secondary antibodies, peroxidase-conjugated goat anti-mouse Ig Fc and goat anti-human Ig Fc, were diluted 1:5,000 and 1:10,000 (Sigma-Aldrich), respectively. A positive result was recorded when the difference in OD between test and control wells was greater than 0.2. The results are presented as relative binding defined as the reactivity of mAb or human serum to mutant VLPs divided by the reactivity of the same mAb or human serum observed with wild-type VLPs. This calculation was performed separately for each monoclonal antibody. The data presented are the means of three determinations.

### Monoclonal antibodies, polyclonal antibodies and human sera

A pool of serum from mice immunized with MCPyV MKT21 VLPs was used to evaluate cross-reactivity among polyomaviruses and to validate the neutralization assay with MCPyV pseudovirions [25].

The type-specific anti-BKV mAb BKPyV-6A2 and anti-SV40 mAb SV40-10C5 produced previously were also investigated for their capacity to bind to MCPyV VLPs (unpublished data).

Ten human sera were obtained from MCC patients from the Dermatology Department of Tours University Hospital (Tours, France)[21]. The protocol had been approved by the Comité de Protection des Personnes, Tours-Région centre Ouest1 (ID: 2009-A01056-51).

## Results

### Generation of MCPyV VP1 insertional mutants and anti-MCPyV VP1 monoclonal antibodies

Generation of the insertion mutant of the major capsid protein has been shown to be a valid approach to identify major conformational epitopes located on external antigenic loops [43,44]. The published cristal structure of MCPyV confirmed our insertion sites at the tip of BC, DE, EF and HI loops [23].

VP1-StreptagII insertion mutants were expressed using recombinant baculoviruses and purified from insect cell nuclei using isopycnic banding in CsCl gradient. Fractions with a density around 1.272 g/ml presented a protein of 45 kDa (data not shown).

**Fig 1:**
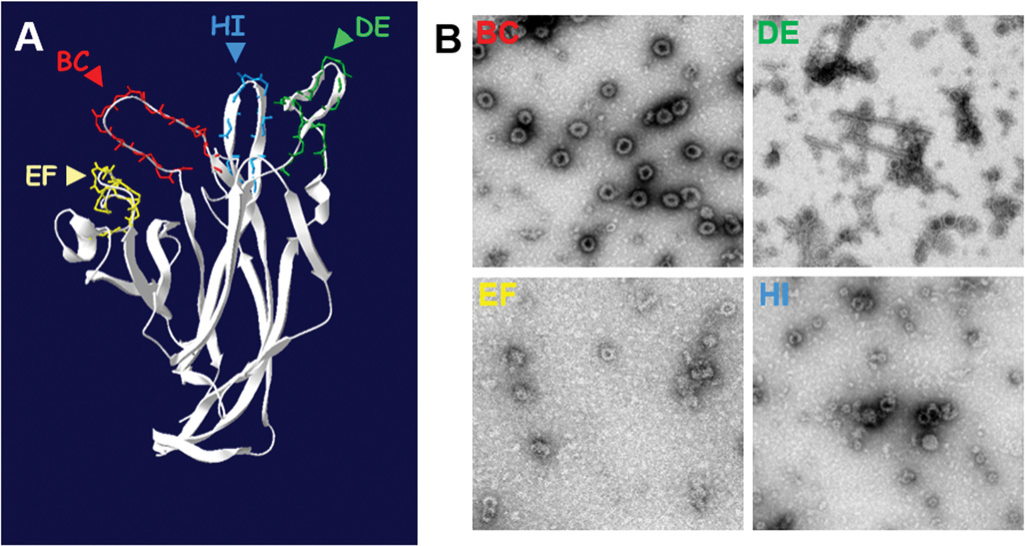
MCPyV VP1 insertional mutants BC, DE, EF and HI. **A)** MCPyV surface exposed loops model generated using the MKT21 sequence with the VP1 structure information of MCPyV w162 strain (4FMG pdb file) by Swiss-Model. The StreptagII motif (WSHPQFEK) coding sequence was inserted into each predicted surface exposed loop, after S88 of BC, after H150 of DE, after T189 of EF and after T296 of HI to generate four insertional mutants, BC, DE, EF and HI, respectively. **B)** MCPyV VP1 mutant particles observed by transmission electron microscopy after recombinant baculovirus expression and CsCl gradient purification.

Electron microscopy observation of these fractions indicated that the VP1 protein of the four mutants assembled into VLPs. However, DE, EF and HI VP1 mutants were more irregular in shape and size than the BC VP1 mutant which was indistinguishable from wt VLPs (Fig. 1). Exposure of the StreptagII motif at the surface of native mutant VLPs was confirmed for the four mutants using an anti-StreptagII monoclonal antibody (data not shown).

Anti-MCPyV VP1 mAbs were generated using both MCC350 VP1 which have been shown not to self-assemble into VLPs but only to form VP1 aggregates and MKT21 VP1 which self-assembled into VLPs [25].

Sixty-five MCC350 hybridoma clones were generated, of which seven anti-VP1 secreting hybridomas were characterized. In addition, sixty-nine hybridomas were generated against MKT21 VP1 that self-assembled into VLPs, of which seven anti-VP1 secreting hybridomas were characterized. Overall, twelve of these mAbs were IgG and two anti-MKT21 VLPs were IgM (MKT21-3D2 and MKT21-3D3) (Table 2).

**Table 2:** Characteristics of 14 anti-MCPyV mAbs produced with VP1 from MCC350 and MKT21 strains. BL, buried linear, C, conformational.

Name	Ig isotype	Type of epitope	PyV Type specificity	Neutralization
MCC350-A1B	IgG2a	BL	ND	-
MCC350-D5D	IgG3	BL	ND	-
MCC350-B4A	IgG3	BL	ND	-
MCC350-D1D	IgG3	BL	ND	-
MCC350-C1A	IgG2a	BL	ND	-
MCC350-B1C	IgG3	BL	ND	-
MCC350-B6B	IgG3	BL	ND	-
MKT21-1D1	IgG3	C	MCPyV	*+*
MKT21-1D3	IgG3	C	MCPyV	*+*
MKT21-1D4	IgG2a	C	MCPyV	+
MKT21-3D2	IgM	C	MCPyV	+
MKT21-3D3	IgM	C	MCPyV	+
MKT21-5D2	IgG2a	C	MCPyV	+
MKT21-6A6	IgG3	C	MCPyV	+
Anti-BKPyV	ND	C	BKPyV	ND
Anti-SV40	ND	C	SV40	ND
MKT21 polyclonal	ND	ND	MCPyV, SV40, LPyV, BKPyV	+

### Characterization of anti-MCPyV VP1 monoclonal antibodies

The reactivity of the 14 anti-VP1 mAbs was investigated against intact and disrupted MKT21 VLPs. Antibodies reacting with intact but not disrupted VLPs were considered to be directed against conformational epitopes located on the exposed surface of the viral capsid. Persistent reactivity after disruption of the VLPs was interpreted as evidence of exposed linear epitopes at the surface of the viral capsid. In the case of reactivity only to disrupted VLPs, this was taken as evidence of buried linear epitopes. All the mAbs generated with MCC350 VP1 recognized buried linear epitopes. In contrast, all mAbs generated using MKT21 VLPs bound to conformational epitopes (Table 2).

Cross-reactivity of the seven conformation-dependent mAbs was investigated with other human polyomavirus VP1 VLPs (SV40, LPyV, HPyV6, HPyV7, HPyV9, TSPyV and BKPyV). None of them exhibited cross-reactivity. It should be noted that two previously generated monoclonal antibodies, BKPyV-6A2 and SV40-10C5 mAbs, were also type-specific and conformation-dependent (Table 2).

The anti-MKT21 monoclonal antibodies were also able to neutralize MCPyV pseudovirions in contrast to the seven antibodies generated against MCC350 VP1. As our aim was to identify conformational neutralizing epitopes, the subsequent experiments were performed only with the MKT21 derived mAbs.

**Fig 2:**
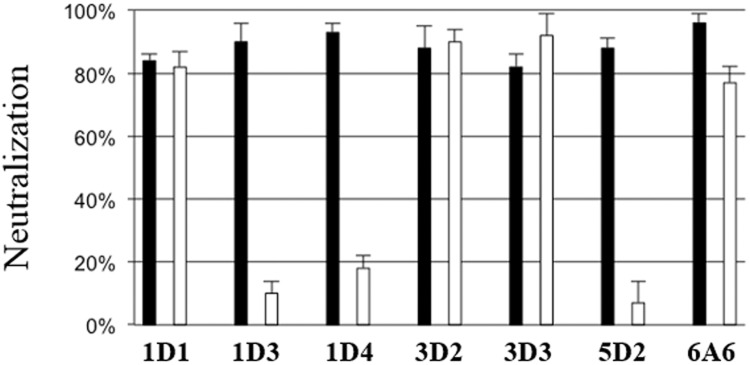
Monoclonal antibodies neutralization mechanism after pre (black) or post (white) MCPyV pseudovirions attachment. For the detection of neutralizing antibodies, COS-7 cells (10^4^/well) and MCPyV luciferase pseudovirions (0.2 RLU) were used. For the pre attachment determination, pseudovirions were mixed with monoclonal antibodies supernatants diluted 1:3 during 1 h and then added to the cells for 3 h at 37°C. The mixture was removed and 100 μl of DMEM-FCS were added. For investigation of post-attachment neutralization, pseudovirions were bound to cells for 1 h at 4°C. Unbound virions were removed and then antibodies diluted 1:3 were added during 1h. The antibodies were removed and 100 μl of DMEM-FCS were added. After incubation for 48 h at 37°C the luciferase activity was measure. The results were expressed as the percentage of inhibition of luciferase activity. The data presented are the means of three determinations performed in duplicate (+/- SEM).

To determine whether the neutralization took place before or after attachment of the pseudovirions to cells the seven neutralizing mAbs were added pre and post attachment and then the reduction of transduction efficiency was determined. Experiments dichotomized the neutralizing feature of the mAbs. MKT21-1D3, MKT21-1D4, and MKT21-5D2 neutralized pseudovirions before their attachment to cells, whereas MKT21-1D1, MKT21-3D2, MKT21-3D3, and MKT21-6A6 mAbs neutralized the pseudovirions after cell binding (Table 2, Fig. 2).

### Location of MCPyV VP1 neutralizing immunodominant epitopes

**Fig 3:**
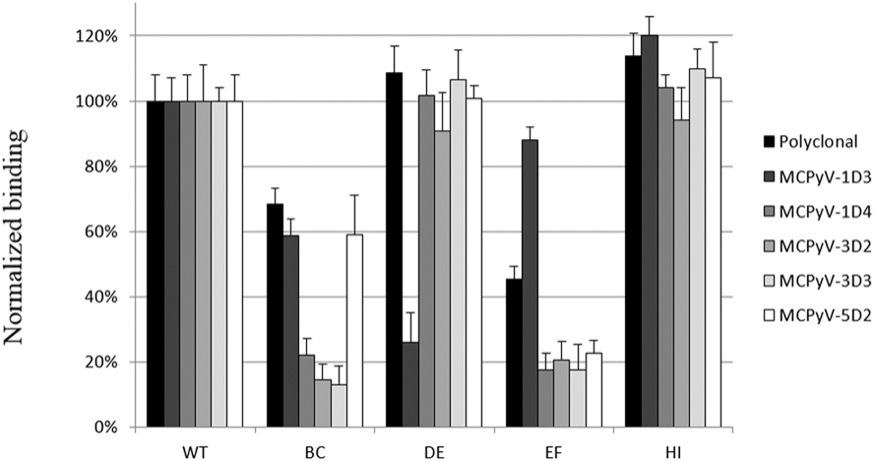
ELISA reactivity of MCPyV monoclonal antibodies against MCPyV insertional mutants BC, DE, EF and HI. In order to characterize the epitopes of the MCPyV VP1, the reactivity of mAbs was analyzed using the MCPyV wt VP1 VLPs and the four mutants with insertion within the BC, DE, EF and HI VP1 loops. ELISAs were performed using hybridoma culture supernatants diluted 1:3. The results are presented as relative binding defined as the reactivity of mAb to mutant VLPs divided by the reactivity of the same mAb observed with wild-type VLPs. The data presented are the means of three determinations (+/- SEM).

The reactivity of the five mAbs exhibiting the highest neutralizing activity (MKT21-1D3, MKT21-1D4, MKT21-3D2, MKT21-3D3 and MKT21-5D2) was evaluated against wild type and the four MCPyV VP1 mutants (Fig. 3). The results indicated that insertion of heterologous sequences into the DE and HI loops did not affect reactivity of any of the five mAbs investigated, with the exception of the MKT21-1D3 mAb where a strong reduction in reactivity observed against a DE mutant. In contrast, the reactivity of the five mAbs was dramatically reduced with the EF mutant. The reactivity of three mAbs, MKT21-1D4, MKT21-3D2 and MKT21-3D3, was dramatically reduced with the BC mutant, but only a moderate reduction in reactivity was observed with MKT21-1D3 and MKT21-5D2 mAbs.

**Fig 4:**
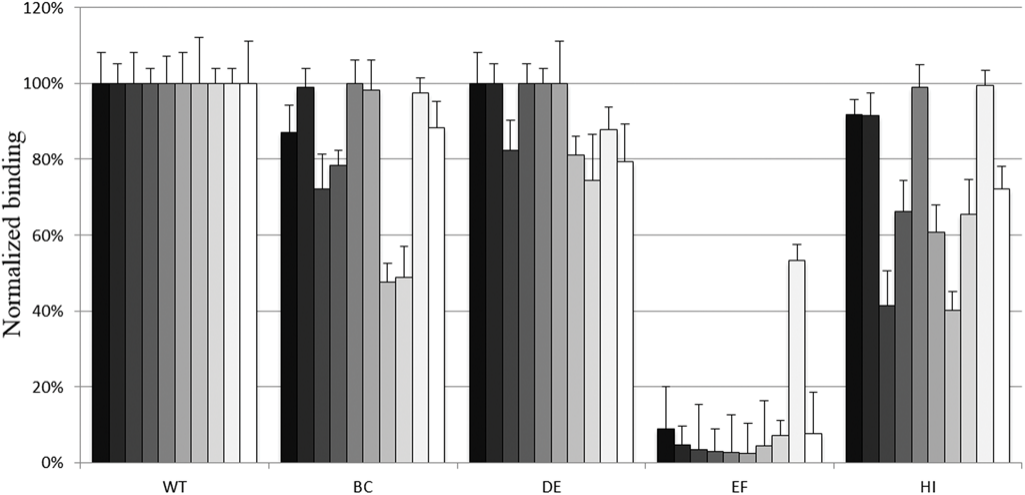
ELISA reactivity of 10 neutralizing anti-MCPyV positive human sera (MCC patients) against MCPyV insertional mutants BC, DE, EF and HI. In order to characterize the epitopes of the MCPyV VP1, the reactivity of the human sera was analyzed from ten patients using the MCPyV wt VP1 VLPs and the four mutants with insertion within the BC, DE, EF and HI VP1 loops. ELISAs were performed using neutralizing anti-MCPyV positive human sera diluted 1:1000. The results are presented as relative binding defined as the reactivity of human serum to mutant VLPs divided by the reactivity of the same human serum observed with wild-type VLPs. The data presented are the means of three determinations (+/- SEM).

In addition, the reactivity of the four mutants against ten anti-MCPyV positive human sera was also evaluated. The neutralizing capacity of all the 10 human sera was first revealed using the MCPyV pseudovirions (data not shown). Reactivity of all the human sera investigated was dramatically reduced when tested against the EF loop insertion mutant and to a lesser extent against the HI and BC mutants (Fig. 4).

## Discussion

The strategy adopted to map immuno-dominant epitopes of the MCPyV VP1 capsid protein is based on the assumption that the hypervariable loops at the capsomer surface contain conformational neutralizing epitopes, and that insertion of small amino acid sequences abrogates antibody binding, similarly to the approach used for mapping conformational epitopes of papillomaviruses and other polyomaviruses [31,33,43,45]. Insertion sites were initially determined by using an MCPyV VP1 MKT21 model constructed using Swiss model and SV40 VP1 cristallographic data as template (1SVA). The MCPyV VP1 w162 strain crystallographic data were then available [23], and no major variation in the loops were observed between the experimental and predicted models.

As observed for other polyomaviruses and papillomaviruses, insertion of short peptide sequences within the hypervariable loops did not dramatically alter the structure of the VLPs [43,46]. VLPs derived from DE, EF and HI mutants exhibited a lower rate of T = 7 symmetry VLPs associated with the presence of smaller VLPs. However, this heterogeneity is also observed in wild type VLPs from MWPyV, HPyV6, HPyV9 and TSPyV [28,38].

In order to characterize the antigenic region of the MCPyV capsid, a panel of anti-VP1 mAbs was produced. The mAbs produced using MCC350 VP1 recognized only buried linear epitopes, possibly explained by the fact that this protein did not assemble into VLPs [25]. All mAbs developed against MKT21 VP1 were conformation-dependent and type-specific. This was also observed by Pastrana et al. using VLPs derived from the MCC 339 sequence [30]. It could be noticed that our panel is less rich in IgM clones, probably due to the fact that our immunization schedule included a booster dose.

All the conformation-dependent mAbs exhibited neutralizing activity against MCPyV pseudovirions, confirming the findings of Pastrana et al. [30], and similar to those of Randhawa et al. with BKPyV [47]. Pre- and post-attachment neutralization experiments indicated that three out of the seven mAbs did not retain their neutralizing activity when added after attachment. This suggests a conformational change of the capsid that occurred after the binding of the viral particles to a primary cell receptor, as observed for papillomaviruses [34,48–50].

Using MCPyV-specific mAbs and the VP1 insertion mutants allowed us to identify two immunodominant epitopes within the BC and EF loops. The determinants of a conformational epitope may be non-contiguous along a polypeptide sequence and occur in spatial proximity only when the protein is folded. The finding that the two VP1 surface loops BC and EF contributed to the immunodominant neutralizing epitope of MCPyV is consistent with the findings of Murata et al. [35] identifying neutralization epitopes of SV40 on the same hypervariable loops. The presence of a conformational epitope on the BC loop has also been reported for mPyV [46,51], and suggested for BKPyV, since serological subtypes are determined by mutations within the BC loop [52]. Neutralizing antibodies presumably target functional domains on the capsid domains. It was previously shown that sialylated glycans can act as primary receptors for MCPyV [53] and that the binding to sialic acid was attributed to amino acids located on the BC loop [23]. These results together suggest that the BC loop plays a key role in virus entry and is the main target for immune response against MCPyV.

Analysis of the reactivity of the MCC patient sera with the four MCPyV mutants indicated that the BC and HI mutants were less reactive to serum from some patients whereas the EF mutant was poorly recognized by all MCC patient sera. These findings suggested that a major antigenic region is located within the EF loop as did those obtained with monoclonal antibodies.

In conclusion, our findings indicate the existence of type-specific conformational neutralizing epitopes on the MCPyV VP1 capsid on BC and EF loops, in agreement with the involvement of at least the BC loop in cell binding. In addition, neutralization was observed with some of the mAbs only before cell binding, suggesting a conformational change in the pseudovirions before cell entry, as observed for Papillomaviruses [40,48].
